# Reduced proviral loads during primo-infection of sheep by Bovine Leukemia virus attenuated mutants

**DOI:** 10.1186/1742-4690-1-31

**Published:** 2004-10-05

**Authors:** Christophe Debacq, Maria Teresa Sanchez Alcaraz, Franck Mortreux, Pierre Kerkhofs, Richard Kettmann, Luc Willems

**Affiliations:** 1 Molecular and cellular biology, FUSAGx, Gembloux, Belgium; 2 Unité d'Oncogenèse Virale, CNRS UMR5537, Centre Léon Bérard, Lyon, France; 3 Department of Virology, Veterinary and Agrochemical Research Centre, Uccle, Belgium

## Abstract

**Background:**

The early stages consecutive to infection of sheep (e.g. primo-infection) by Bovine leukemia virus mutants are largely unknown. In order to better understand the mechanisms associated with this period, we aimed at analyzing simultaneously three parameters: B-lymphocytosis, cell proliferation and viral replication.

**Results:**

Sheep were experimentally infected either with a wild type BLV provirus or with selected mutants among which: a virus harboring an optimalized LTR promoter with consensus cyclic AMP-responsive elements, two deletants of the R3 or the G4 accessory genes and a fusion-deficient transmembrane recombinant. Seroconversion, as revealed by the onset of an anti-viral antibody response, was detected at 3 to 11 weeks after inoculation. At seroconversion, all sheep exhibited a marked increase in the numbers of circulating B lymphocytes expressing the CD5 and CD11b cluster of differentiation markers and, interestingly, this phenomenon occurred independently of the type of virus. The net increase of the absolute number of B cells was at least partially due to accelerated proliferation as revealed, after intravenous injection of bromodeoxyuridine, by the higher proportion of circulating BrdU+ B lymphocytes. BLV proviral DNA was detected by polymerase chain reaction in the leucocytes of all sheep, as expected. However, at seroconversion, the proviral loads were lower in sheep infected by the attenuated proviruses despite similar levels of B cell lymphocytosis.

**Conclusions:**

We conclude that the proviral loads are not directly linked to the extent of B cell proliferation observed during primo-infection of BLV-infected sheep. We propose a model of opportunistic replication of the virus supported by a general activation process of B lymphocytes.

## Background

Bovine leukemia virus (BLV) is an oncogenic retrovirus closely related to the primate T-cell leukemia viruses [1]. These viruses are exogenous to their host species [2,3], have similar genomic organizations [4], integrate into dispersed sites within the host genome [5,6] and appear transcriptionally silent in vivo (reviewed by [7]). However, BLV is unique in the HTLV family of retroviruses because it infects and dysregulates B lymphocytes instead of T cells. The natural host for BLV is cattle but the virus can also be experimentally transmitted to sheep [8]. The pathogeneses in these species are globally similar despite higher frequencies of leukemogenesis in sheep (up to 100%) and shorter latency periods (1–4 years versus 4–10 in cattle) [1]. Following BLV infection, the hosts, either cattle or sheep, develop a persistent antibody response to viral proteins and virions can be isolated from ex vivo cultured leucocytes [9]. Detection of antibodies in the infected animals correlates with a transient B cell lymphocytosis [10-13]. In most cases, BLV infection remains clinically silent, a stage referred to as the asymptomatic or aleukemic stage of the disease [7]. Only 30% of BLV-infected cattle develop persistent lymphocytosis (PL), a polyclonal expansion of B cells coexpressing high levels of surface IgM, myeloid (CD11b) or T-specific (CD5) markers [14-16] and less than 5% will die from a fatal leukemia, lymphoma or lymphosarcoma [17].

A major advantage of the BLV system is the possibility to study viral genetic determinants in relation with infectivity and pathogenicity in vivo. A strategy, which we previously described, is based on the use of a cloned BLV provirus whose sequence can be mutagenized in vitro. Well characterized mutants can subsequently be injected into sheep and compared to the wild type virus (WT) [18]. This experimental protocol permitted the correlation of viral determinants with defined phenotypes in vivo. In particular, we showed that:

(i) the R3 and G4 accessory genes are required for efficient viral spread in vivo, although their deletion or mutation does not hamper infectivity (mutants CRX3 and IG4 described by [1,19,20]).

(ii) restoring a CRE consensus (cyclic-AMP response element; TGACGTCA) in the triplicate motif of the imperfectly conserved Tax-responsive sequence (TxRE: AGACGTCA, TGACGGCA, TGACCTCA) increases LTR promoter activity, as expected, but restricts the proviral loads in vivo, suggesting that repression of expression is required for immune escape (CRE mutant; [21]).

(iii) formation of multinucleated syncytia by envelope dependent cell fusion in vitro is paradoxically not required for infectivity or efficient viral spread in vivo (A60V recombinant; [22]).

Importantly, only the A60V envelope mutant behaves as wild type in terms of infectivity and pathogenesis, in contrast to the others (CRX3, IG4 and CRE) therefore referred to as attenuated. The two categories of viruses, either wild type (WT and A60V) or attenuated (CRX3, IG4 and CRE) thus permit to characterize and compare the processes occurring during primo-infection.

## Results

### The extent of transient lymphocytosis is similar in sheep infected by wild type or mutant proviruses

**Figure 1:**
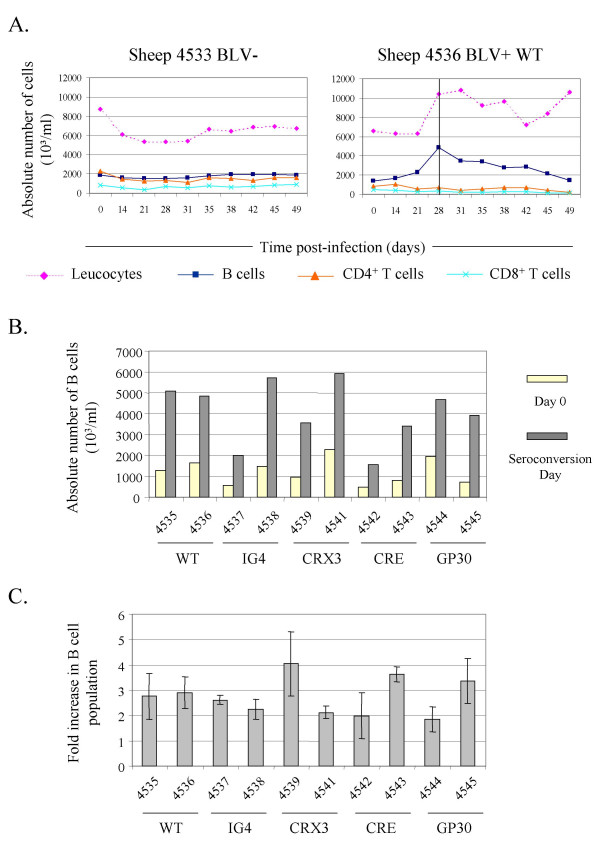
**Transient B cell lymphocytosis at seroconversion of sheep infected by BLV-mutants. **A. Sheep n° 4536 was injected with 100 μg of proviral DNA (wild type strain 344 cloned into plasmid pBLV344) whereas animal n° 4533 was used as a negative control. At different times post-injection, leucocytes were counted using a Coulter Counter ZN and the total numbers of lymphocytes were estimated after examination under a microscope. In parallel, Peripheral Blood Mononuclear Cells (PBMCs) were isolated by Percoll gradient centrifugation and the proportions of B, CD4^+ ^T and CD8^+ ^T-cells were determined by flow cytometry. The vertical line represents the detection by immunodiffusion of antibodies directed against the virus (day 28: sheep n° 4536). B. Graphic representation of absolute B cell numbers at viral inoculation (Day 0, yellow bars) and at seroconversion (grey bars) of sheep infected by the BLV wild type virus (WT) or mutants (IG4, CRX3, CRE and GP30). The sheep coordinates are indicated. C. Fold increase of the B cell population in the infected sheep. The bars correspond to the mean difference (± standard deviation) between the absolute numbers of B cells at the day of virus inoculation and those determined at seroconversion (means of three values).

BLV recombinants with optimized consensus CRE sequences (CRE clone) and mutants deleted in the R3 and G4 genes (CRX3 and IG4 proviruses) are impaired in their ability to propagate efficiently within their host [1,19,21]. The early stages occurring soon after infection by these mutants and, more particularly, the extent of the transient B lymphocytosis are however unknown. Therefore, sheep were infected with well-characterized molecular clones of BLV proviruses. As control, the hematological parameters were first determined in uninfected animals, as illustrated on figure 1A (sheep 4533 BLV-). To this end, PBMCs (Peripheral Blood Mononuclear Cells) were isolated and analyzed by flow cytometry for the presence of IgM, CD4 and CD8 markers. Despite some variations in the absolute numbers of leucocytes, the B (squares), CD4^+ ^(triangles) and CD8^+ ^(crosses) T cell populations remained remarkably constant over extended periods of time, as expected (Figure 1A and data not shown for sheep 4534). In contrast, a marked increase of the absolute numbers of leucocytes occurred between 2 and 3 weeks post-inoculation of the wild type BLV provirus (from 6,331 10^3^/ml to 10,262 10^3^/ml, respectively, at days 21 and 28 in sheep 4536 BLV+ WT, Figure 1A; and data not illustrated for animal n° 4535).

Phenotypic analysis of the sheep n° 4536 PBMCs revealed that leucocytosis is essentially due to a marked increase in the B cell numbers (from 2,294 10^3^/ml of blood to 4,858 10^3^/ml, respectively, at days 21 and day 28; see squares), while the absolute counts of CD4^+ ^(triangles) and CD8^+ ^(crosses) T cells remained relatively constant (as did the γδ T cell population, data not shown). Furthermore, maximal B cell accumulation corresponded to the day of seroconversion characterized by the onset of an anti-BLV humoral response (the vertical line at day 28 on Figure 1A).

A similar experiment was performed in parallel with 4 selected BLV mutants (IG4, CRX3, CRE and GP30), the latter behaving as wild type in terms of pathogenesis and infectivity in vivo. Interestingly, B cell lymphocytosis occurred in all sheep independently of the type of provirus (Figure 1B, compare absolute numbers of B cells at day of seroconversion). A rate of induction was calculated by dividing the absolute numbers of the B cell population around seroconversion with those measured at the day of proviral injection (day 0). This induction rate (represented in fold increase on Figure 1C) was independent of the type of provirus, no significant difference being observed between the wild type and the mutants.

**Figure 2:**
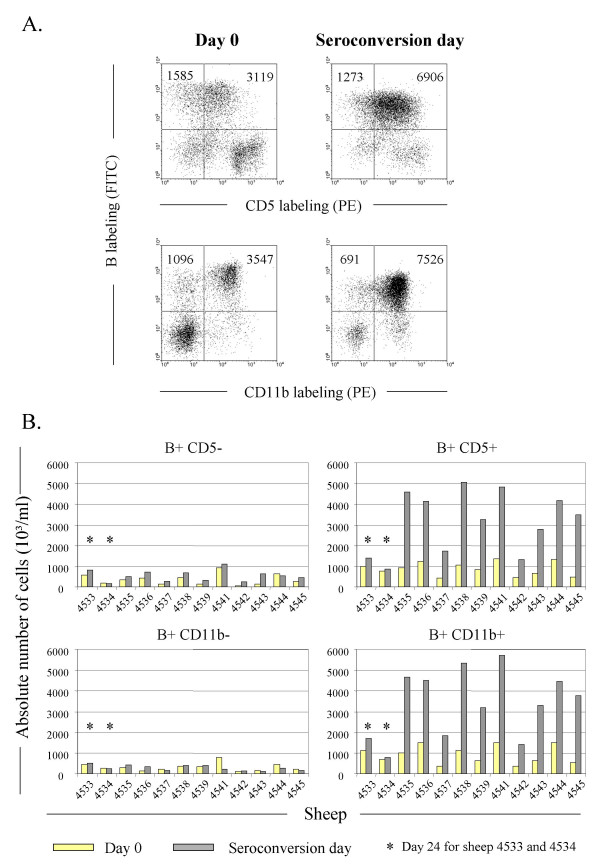
**The lymphocytosis is due to an accumulation of B lymphocytes expressing CD5 and /or CD11b. **A. PBMCs were isolated from BLV wild type infected sheep (n° 4535) and labeled with anti-sIgM 1H4 monoclonal in combination with CD5 or CD11b antibodies. Ten thousand cells analyzed by flow cytometry are represented as dot plots (Y axis = B cells; X axis = CD5 or CD11b expressing cells). Illustrated data correspond to the dot plots performed at day 0 (provirus injection) and at the seroconversion day. The total numbers of B cells are indicated in the upper quadrants. B. Histogram representation of the absolute numbers of B cells expressing (right panels) or not (left panels) CD5 or CD11b in sheep infected with wild type viruses (n° 4535 and 4536), or mutants (pBLVIG4 in n° 4537 / 4538, pBLVCRX3 in n° 4539 / 4541, pBLVCRE3X in n° 4542 / 4543, pBLVA60V in n° 4544 / 4545). Sheep n° 4533 and 4534 were used as uninfected controls. * represents the absolute number of cells at day 24 of the experiment.

Expression of the CD5 and CD11b cluster of differentiation markers has been associated with BLV-infection, although B lymphocytes negative for these receptors are also less efficient targets for the virus [23,24]. Therefore, we aimed to determine if lymphocytosis detected at the seroconversion period is preferentially due to an accumulation of B+CD5+ and/or B+CD11b+ cells. For this purpose, PBMCs were dually labeled with an anti-IgM antiserum together with anti-CD5 or anti-CD11b monoclonal antibodies and analyzed by two-color flow cytometry (illustrated on Figure 2A for BLV wild type infected animal n° 4535: x axis = CD5 or CD11b labeling; y axis = B labeling). At day 0, two third of the B lymphocytes expressed CD5 (3,119 in a total of 1,585 + 3,119 B cells) whereas, at seroconversion, most of them became positive for this marker (6,906 cells versus 1,273). In parallel, the number of B+CD11b+ cells also drastically increased (3,547 cells at day 0 versus 7,526 at the seroconversion day). Of note, although most B lymphocytes should be CD5 and CD11b double positive cells, this assumption could not be formally demonstrated because of cross reactions between two IgG1 isotypes. However, the dot plots at right on the figure 2A clearly show that the majority of B cells should express both markers.

In terms of absolute cell counts (i.e. normalized to the cell numbers per ml of blood), it appeared that B+CD5+ or B+CD11b+ lymphocytes accumulated at the seroconversion day in wild type infected sheep 4535 and 4536 (compare yellow and shaded bars on the right panels of Figure 2B). This accumulation did not happen in the CD5- or CD11b-negative B cell populations (left panels) or in negative controls (uninfected sheep n° 4533 and 4534), although a relative increase was observed in some infected animals (particularly 4542 and 4543, Figure 2B, B+CD5-). Importantly, the net increase of sIgM+CD5+ or sIgM+CD11b+ populations also occurred in animals infected by the different mutants independently of the type of provirus (Figure 2B).

Together, these data demonstrate that primo-infection in sheep experimentally infected either with a wild type or mutant BLV proviruses is associated with a transient lymphocytosis, extending previous reports [11,12]. At seroconversion, all sheep exhibited a marked increase in the numbers of circulating B lymphocytes expressing for most of them the CD5 and CD11b cluster of differentiation markers and, interestingly, this phenomenon occurred independently of the type of mutant.

### Increase of in vivo B cell proliferation during seroconversion

In terms of cell dynamics, lymphocytosis is the consequence of a homeostatic deregulation provoked by an imbalance in the rates of proliferation and/or death. Alternatively or concomitantly, the net increase in B lymphocyte numbers might be the result of a cell mobilization between the lymphatic system and the peripheral blood. We have previously established a protocol allowing to quantify these parameters in BLV-infected sheep [25]. This experimental approach is based on intravenous injection of 5-bromo-2'-deoxyuridine (BrdU) which permits, after its incorporation into DNA, the identification by flow cytometry of cells that have undergone proliferation.

**Figure 3:**
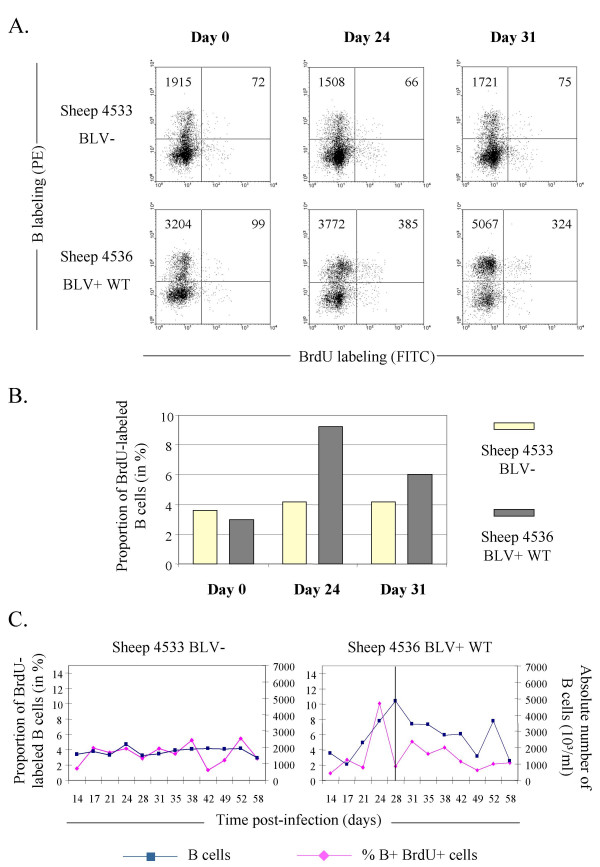
**The lymphocytosis correlates with increased B cell proliferation at seroconversion. **Once per week over a 2 months period, 500 mg of bromodeoxyuridine (BrdU) were injected intravenously into BLV-infected (n° 4536) and control (n° 4533) sheep. An aliquot of blood (1 ml) was collected at three days post-BrdU injection. After lysis of the red blood cells, B lymphocytes were labeled with an anti-sIgM antibody. Next, cells were stained with anti-BrdU FITC in the presence of DNase and analyzed by two-color flow cytometry. A. Dot Plot graphs of B lymphocytes (Y axis) labeled in combination with BrdU (X axis) in uninfected control animal (n° 4533) and in a BLV wild type infected sheep (n° 4536) performed at days 0, 24 and 31. Day 31 corresponds to the first data obtained just after seroconversion of sheep n° 4536. Ten thousand events were acquired by flow cytometry and PBMCs were selected by the FSC/SSC gating method. The total numbers of B cells are indicated in the upper quadrants. B. Histogram of the proportions of BrdU+ labeled B cells (in % of the total B lymphocyte population). C. Graphic representation of the proportions of B+BrdU+ cells within the total B lymphocyte population (at days 3 and 7 post-injection) and the corresponding absolute numbers of B lymphocytes observed during the experiment. The BrdU was injected at days 14, 21, 28, 35, 42 and 52. The vertical line represents the detection of the seroconversion day.

In order to label proliferating B lymphocytes during primo-infection, BrdU was injected weekly over a two months period and blood was collected at three days after each injection, a delay required for maximal detection of labeled cells (see methods) [25]. Figure 3A illustrates an example of IgM+BrdU+ dual flow cytometry analysis performed at days 0, 24 and 31 after proviral injection into sheep n° 4536. It appeared that the numbers of B+BrdU+ cells in this sheep increased at day 24 (cell counts of 99 at day 0 versus 385 at day 24 amongst 10,000 events) and remained high at day 31 (324 counted events). In contrast, in sheep n° 4533 used as a negative control, no variation was observed in terms of absolute numbers (72, 66 and 75 at days 0, 24 and 31) as well as in proportion of the sIgM-positive cell population (Figure 3B, yellow bars). In BLV-infected sheep n° 4536, however, this relative proportion of BrdU-labeled B lymphocytes peaked at day 24, indicating that these cells underwent proliferation (black bars on Figure 3B). Interestingly, the burst of B+BrdU+ cells just preceded the onset of seroconversion (vertical line at day 28 on Figure 3C) and correlated with the mid phase of total B lymphocytes counts in the blood. The most straightforward interpretation is that the net increase of the B cell population is at least partially due to proliferation.

**Figure 4:**
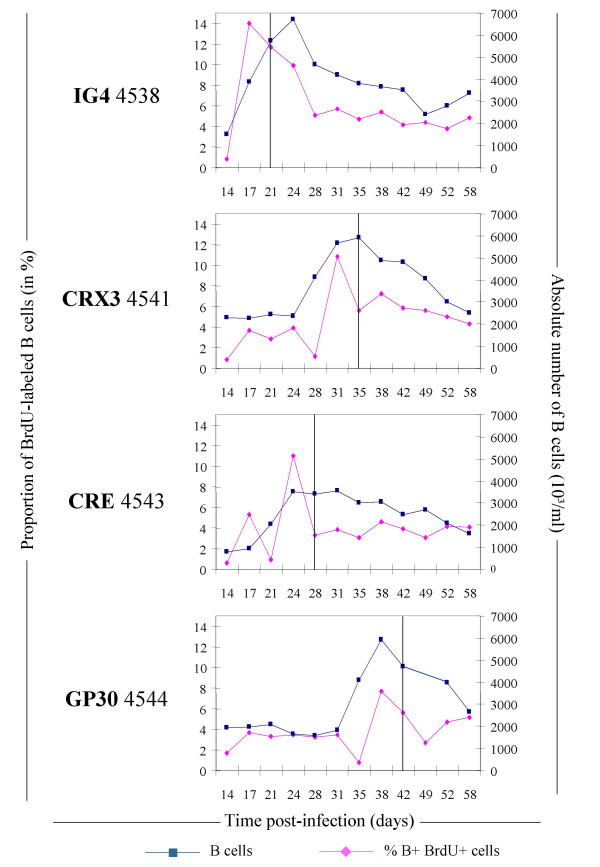
**In vivo B lymphocyte proliferation in sheep infected with BLV mutant proviruses. **Graphic representation of the proportions of B+ BrdU+ cells within the total B lymphocyte population (at days 3 and 7 post-injection) and the corresponding absolute numbers of B lymphocytes in sheep (n° 4538, 4541, 4543 and 4544) infected, respectively, with BLV mutants (IG4, CRX3, CRE and GP30). The BrdU was injected at days 14, 21, 28, 35, 42 and 52. The vertical line corresponds to the seroconversion day as determined by an immunodiffusion test.

Since BLV mutants also induce a transient lymphocytosis, a kinetics of BrdU incorporation was performed in 4 additional sheep infected with proviruses IG4, CRX3, CRE and GP30 (respectively animals n° 4538, 4541, 4543 and 4544; see figure 4). In all sheep, the high absolute numbers of B lymphocytes (squares) observed at seroconversion (vertical line) was preceded by a net increase in the BrdU positive population (lozenges).

Together these data demonstrate that transient lymphocytosis occurring in BLV infected-sheep is at least partially due to an increase in cell proliferation as assessed by BrdU incorporation and, surprisingly, this phenomenon occurred independently of the type of virus.

### Lymphocytosis and B cell proliferation are independent of the proviral loads

**Figure 5:**
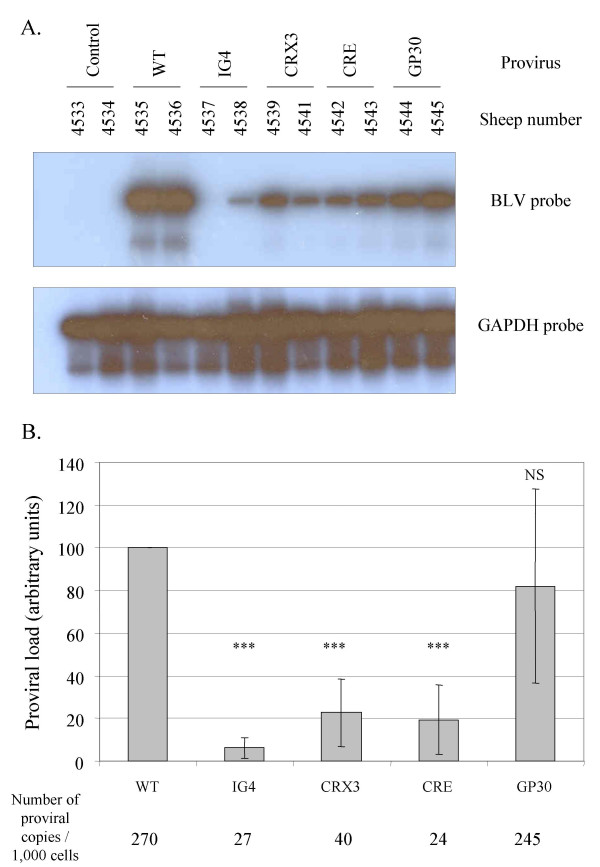
**Reduced proviral loads at seroconversion in sheep infected with attenuated mutants. **A. At seroconversion day, DNA was extracted from blood isolated from wild type or mutant-infected sheep, as indicated. Proviral sequences were amplified by 25 cycles of PCR using *tax *specific primers. The amplification products were resolved on a 1% agarose gel and analyzed by Southern blotting with a BLV *tax *probe. Blood samples from uninfected sheep n° 4533 and 4534 were used as controls for PCR contamination. A PCR amplification of the *gapdh *gene was used as an internal control for DNA integrity. B. Mean proviral loads at seroconversion. For each category of mutant-infected sheep, the mean values of the proviral loads (in arbitrary units ± standard deviations as determined after *Phosphorimaging *scanning) were statistically compared using the Student *t *test to the mean proviral load of the wild type group (WT). The data result from three independent experiments using DNAs extracted around the seroconversion period (NS: p > 0.05 non-significance; * 0.01 < p < 0.05; *** p < 0.001, Student *t *test). Using real-time PCR, the proviral loads were estimated to be 270 and 245 copies per 1,000 cells in sheep infected, respectively, with the wild type virus and the GP30 recombinant whereas the levels yielded by the other mutants were significantly reduced (27, 40 and 24 copies / 1,000 cells for IG4, CRX3 and CRE, respectively).

With the aim to quantify the proviral loads, viral DNA levels within the circulating blood of all sheep were determined by semiquantitative PCR. The genomic DNAs were extracted from blood samples collected at seroconversion and the sequences corresponding to the viral *tax *gene were amplified by PCR. The number of PCR cycles was adapted in order to compare the relative amounts of proviral sequences in the blood samples. The amplicons were then analyzed by Southern Blotting using a BLV *tax *probe. Amplification of the *gapdh *gene was used as internal control for chromosomal DNA integrity (Figure 5A). As controls for PCR contaminations, no signal was generated in DNA samples from uninfected sheep at day 0 (n° 4533 and 4534). In contrast, PCR amplification of the *tax *gene using the genomic DNA from WT infected sheep (sheep n° 4535 an 4536) yielded a 1 kb fragment as expected. Under similar conditions, a weaker signal was generated by amplification of genomic DNA isolated from animals infected by mutant proviruses (Figure 5A). Phosphorimager quantification of these hybridization signals revealed that mutants IG4, CRX3 and CRE replicated at lower proviral loads compared to the wild type or the non-attenuated GP30 viruses (Figure 5B). Using real-time PCR performed as described by [26], the proviral loads were estimated to be 270 and 245 copies per 1,000 cells in sheep infected, respectively, with the wild type virus and the GP30 recombinant whereas the levels yielded by the other mutants were significantly reduced (27, 40 and 24 copies / 1,000 cells for IG4, CRX3 and CRE, respectively) (Figure 5B).

Together these data show that, around the seroconversion period, the proviral loads are reduced in sheep infected by attenuated mutants.

## Conclusions

**Figure 6:**
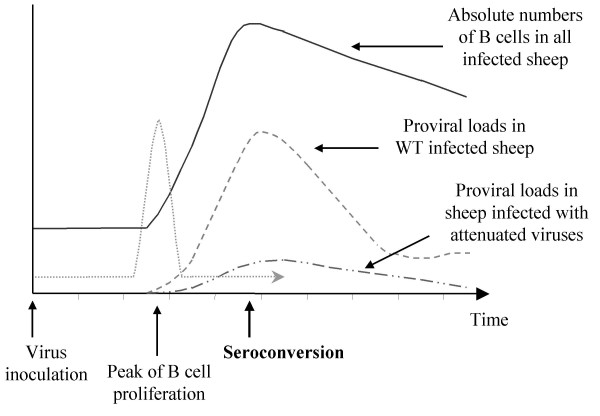
**Schematized summary of proliferation rates, proviral loads and lymphocytosis associated with primo-infection. **Sheep, which were experimentally infected with BLV wild type or mutant proviruses (virus inoculation), exhibited a similar extent of transient accumulation of B-lymphocytes (—). This transient lymphocytosis arising just before seroconversion is at least partially due to an increase in B cell proliferation (peak of B cell proliferation •••••). In contrast, the proviral loads greatly differ among the two categories of sheep, e.g. infected with wild type (- - -) or attenuated viruses (— ••).

We have studied here the interplay between the efficiency of viral spread, the cellular proliferation within the host and the extent of cell accumulation during the period consecutive to the infection of sheep by bovine leukemia virus. We compared two categories of viruses based on their ability to infect and expand within their host: those behaving as wild type (WT and GP30) or so-called attenuated mutants (IG4, CRX3 and CRE). Experimental infection of sheep with these two types of viruses led to a surprising observation, namely, a similar extent of transient lymphocytosis independently of the proviral loads (see Figure 6). In other words, the total B lymphocyte accumulation within the peripheral blood is not modulated by the amount of viral copies. Another contribution of this report is the demonstration that transient lymphocytosis arising just prior to seroconversion is at least partially due to an increase in B cell proliferation (Figure 6). In fact, since lymphocytes rest within the peripheral blood in the G0/G1 phase of the cell cycle (unpublished data), proliferation occurs in other sites i.e. the bone marrow, spleen, lymph nodes and Peyer's Patches. Therefore, the accumulation of BrdU+ B lymphocytes might also be the consequence of an increased outflow from these sources estimated, in non-infected sheep, at 30 × 10^6 ^cells per gram of lymph node in one hour [27]. Conversely, impaired recirculation to the lymphatic system would also create an imbalance in the B lymphocyte counts (1 g of lymph node receives 1.2 × 10^8 ^cells per hour [27]). Answering to this question would require canulation of lymph nodes allowing the precise quantification of the cellular flows.

With the aim to correlate lymphocytosis with a defined B cell sub-population, we have demonstrated that two surface molecules, CD5 and CD11b, whose expression has been previously associated with late stages of BLV infection [13-15,23,24] are also important markers at the seroconversion period. In human and mice, the B lymphocytes expressing the CD5 and CD11b proteins are referred to as B-1a cells. B lymphocytes that are CD5- and CD11b+ are called B-1b and have a similar function than the B-1a subset [28]. Compared to the conventional B-2 cells (e.g. CD5- CD11b-), the B-1 population exhibits different developmental schemes, phenotypes, antibody repertoires, localization and behaviors. In humans, elevated numbers of B-1 cells have been reported in patients with Sjorgen's syndrome, rheumatoid arthritis, chronic lymphocytic leukemia and AIDS [29-32]. In mice, increased numbers of B-1 cells have been observed in a number of naturally occurring and genetically manipulated strains that develop autoimmune manifestations [33]. B-1 cells are believed to be the major source of natural IgM, a polyreactive and weakly autoreactive antibody, which is produced in the absence of exogenous antigenic stimulation [34]. Consistent with this model, IgM specificities within the B-1 repertoire include phosphorylcholine, phosphatidylcholine, thymocytes, lipopolysaccharide and influenza virus [33]. Interestingly, in sheep, the B-1 population also expands in response to infection with other pathogens like *Trypanosoma evansi *and *Pasteurella haemolytica *[35,36]. In this context, we speculate about an opportunistic mechanism of BLV replication supported by a general activation of B-1a lymphocyte proliferation, which could thus be a primary B-cell humoral response. Successive divisions of B-1a cells would thereby expand the number of potential targets for the virus. During this period, the ability of the virus to colonize new cells would be crucial, a fact that is reflected by the differential proviral loads between the wild type and attenuated viruses.

Differences in the infectious potential of the attenuated viruses are the consequence of mutations in accessory genes (R3 and G4) or in the LTR promoter (CRE) [19,21]. Although the role of R3 is still unknown, its homologue expressed by HTLV-1 (p12) interacts with the IL-2 receptor as well as with calcineurin and is crucial during the initial steps of infection [37-39]. BLV G4 and HTLV-1 p13 bind to the same cellular protein, farnesyl pyrophosphate synthetase [40] but modulate differentially cell transformation in vitro [20,41]. The defect in BLV propagation associated with the CRE mutant relates to its inability to repress basal expression [21]. Optimization of the imperfect CRE enhancer sequences present in the LTR creates a more efficient promoter, as expected, but restricts the proviral loads indicating that transcriptional silencing is required for viral persistence and spread. The reduced capacity of the CRE, G4 and R3 mutants to propagate are thus caused by different genetic defects and data presented in this report further extent our previous observations [1,19,21]. More surprisingly is the replication efficiency exhibited by the GP30 envelope mutant. Unable to induce membrane fusion, at least as measured by classical syncytia formation tests, the GP30 virus propagates at wild type levels [22]. Besides a possible experimental caveat based on the lack of sensitivity of the syncytium assay in vitro, our previous (and most straightforward) hypothesis postulated that viral spread mainly occurred via clonal expansion of the infected lymphocytes with few or non-limiting cell-to-cell transmissions. Figure 5 demonstrates that the proviral loads of the GP30 mutant are at wild type levels even during the early steps of infection, a period thought to be associated with active infection of novel cells. Preliminary inverse PCR amplification data indicate that the number of target cells carrying integrated GP30 or wild type proviruses are not significantly different (F. Mortreux, ongoing work). We thus have to reconsider our interpretation and propose that the syncytium assay does not reflect the infectious potential in vivo. In terms of its biological properties in sheep, the GP30 recombinant should thus be considered as, or close to, wild type at least during primo-infection.

In conclusion, we have characterized here the initial steps consecutive to BLV infection of sheep. We show that this period is characterized by a transient accumulation of CD5+ / CD11b+ B lymphocytes resulting, at least in part, from increased proliferation. Furthermore, the extent of B cell lymphocytosis is not directly linked to the proviral loads reached by the wild type and mutant viruses. On basis of a comparative leukemia approach, these results could be informative for the related human T-lymphotropic viruses.

## Methods

### Experimental animals

Twelve sheep of one year old were kept under controlled conditions at the Veterinary and Agrochemical Research Centre (Machelen, Belgium). Two animals (n° 4533 and 4534) were used as uninfected controls whereas sheep n° 4535 and 4536 were experimentally infected with a BLV wild type cloned provirus (strain 344) [18]. Briefly, 100 μg of plasmid DNA were mixed with 200 μl of N-[1-(2,3 dioleoloxyl)propyl]-N,N,N-trimethylammonium methylsulfate (DOTAP; *Roche Diagnostics*) in 1 ml of HBS (20 mM HEPES-150 mM NaCl, [pH 7.4]) and injected intradermally into the back of each sheep. Plasmids containing the mutant proviruses pBLVIG4 (harboring a stop codon in the G4 open reading frame; [19], pBLVCRX3 (deleted in R3; [1]), pBLVCRE3X (in which the CRE imperfect sequences were mutated to TGACGTCA; [21]) and pBLVA60V (alanine codon 60 of the GP30 transmembrane gene being mutated into valine; [22]) were injected, respectively, in sheep n° 4537 / 4538, n° 4539 / 4541, n° 4542 / 4543 and n° 4544 / 4545. Twice a week, the total leukocyte counts were determined by using a *Coulter *counter ZN, and the number of lymphocytes was estimated after examination under the microscope after staining with May-Grunwald Giemsa. In parallel, the sera from each sheep were analyzed for BLV seropositivity using immunodiffusion and enzyme-linked immunosorbent assay (ELISA) techniques [42].

### Immunophenotyping of sheep

Peripheral blood mononuclear cells (PBMCs) were isolated by Percoll gradient centrifugation and their viability was estimated by trypan blue dye exclusion [43]). PBMCs were labeled with monoclonal antibodies (Mabs) directed against surface immunoglobulin M (anti-sIgMs, clone 1H4, mouse IgG1; Pig45A2, mouse IgG2b), CD4 (ST4, mouse IgG1), CD5 (CC17, mouse IgG1), CD8 (CC58, mouse IgG1) and CD11b (CC125, mouse IgG1) provided by C. Howard (Institute for Animal Health, Compton, United Kingdom) and by I. Schwartz-Cornil (INRA, Jouy-en-Josas, France) or obtained from *VMRD Inc*. Cells were then labeled with a rat anti-mouse IgG1 phycoerythrin (PE)-antibody (*Becton Dickinson Immunocytometry Systems*) or with a goat anti-mouse IgG2b fluorescein isothiocyanate (FITC)-conjugate (*Caltag Laboratories*). Finally, PBMCs were analyzed by flow cytometry on a Becton Dickinson FACScan flow cytometer. Ten thousand events were collected for each sample and data were analyzed with the *Cellquest *software (*Becton Dickinson Immunocytometry Systems*).

### Analysis of 5-bromo-2'-deoxyuridine in vivo

Each week during two months, sheep were injected intravenously with 500 mg of 5-bromo-2'-deoxyuridine (*Sigma Aldrich*) resuspended in physiologic serum (NaCl 0.9%). To evaluate BrdU-incorporation, blood was collected at three and seven days after each BrdU injection. The red blood cells were lysed with 1× FACS *Lysing Solution *(*Becton Dickinson Immunocytometry Systems*), the leucocytes were washed twice with PBS containing 0.5% Bovine Serum Albumin (BSA) (*Sigma Aldrich*) and incubated in the presence of biotinylated 1H4 monoclonal antibody for 30 min at 4°C. Next, the cells were labeled with streptavidin-phycoerythrin (*Becton Dickinson Immunocytometry Systems*) and incubated with 1× FACS *Permeabilizing Solution *(*Becton Dickinson Immunocytometry Systems*). Finally, leucocytes were stained with anti-BrdU FITC antibody in the presence of DNase (*Becton Dickinson Immunocytometry Systems*) and analyzed by flow cytometry.

### Semiquantitative PCR analysis

DNA isolations were performed directly on blood using the *Wizard*^®^*Genomic DNA Purification Kit *(*Promega*). An aliquot of 300 μl of blood were mixed with 900 μl of *Cell Lysis Solution *and incubated for 10 minutes at room temperature. After two washes with the same buffer, the cells were resuspended in 300 μl of *Nuclei Lysis Solution *and incubated for one hour at 37°C. Then, the samples were digested during 15 minutes at 37°C in the presence of 1.5 μl of *RNase Solution*. Proteins were precipitated by adding 100 μl of *Protein Precipitation Solution *to the nuclear lysates. After centrifugation at 13,000 g, the supernatant was mixed with an equal volume of isopropanol, centrifuged and ethanol precipitated. Five hundred nanograms of the purified DNAs were amplified in the presence of 200 μM of deoxynucleotides, 2.5 U of Taq DNA polymerase, and 200 ng of primers. The primers used (PCRTA 5'-CTCTTCGGGATCCATTACCTGA-3' and PCRTC 5'-CCTGCATGATCTTTCATACAAAT-3') encompass the region from position 7999 to 6990 of the BLV *tax *gene [44]. In parallel, the primers G3PDHA (5'-CATGTGGGCCATGAGGTCCACCAC-3') and G3PDHS (5'-GACCCCTTCATTGACCTCAACTACA-3') were used to amplify the *gapdh *gene. The samples were denatured for 5 min at 94°C, and amplified by 25 cycles of PCR (30 s at 94°C, 30 s at 57°C, and 1 min at 72°C). After a final elongation step of 10 min at 72°C, 20 μl of the amplification products were resolved on a 1% agarose gel, transferred to a Hybond N+ membrane (*Amersham Pharmacia Biosciences*), and hybridized either with a BLV *tax *(a 1-kb *ClaI *insert from plasmid pGEM7zfLOR1) or with a *gapdh *probe labeled with α-^32^P dCTP. Quantification of ^32^P signal was performed using a *PhosphorImager *(*Personal Molecular Imager FX System*,*Biorad*).

Real-time PCR was performed using 6FAM-labeled MGB probes specific for the BLV *pol *gene and the 18S ribosomal DNA sequences essentially as described in reference [26].

## List of Abbreviations

BLV: Bovine Leukemia Virus; BrdU: 5-bromo-2'-deoxyuridine; CRE: Cyclic-AMP Response Element; PBMCs: Peripheral Blood Mononuclear Cells; WT: Wild Type.

## Competing Interests

The author(s) declare that they have no competing interests.

## Authors' Contributions

CD carried out the most experimental work and drafted the manuscript. MS and FM performed the sample collections and the determination of the proviral loads. PK was responsible for the sheep studies. RK participated to experimental design and interpretation of data. LW conceived the study, its design and coordination. All authors read and approved the final manuscript
